# Investigating the use of signal detection information in supervised learning-based image denoising with consideration of task-shift

**DOI:** 10.1117/1.JMI.11.5.055501

**Published:** 2024-09-05

**Authors:** Kaiyan Li, Hua Li, Mark A. Anastasio

**Affiliations:** a University of Illinois Urbana-Champaign, Department of Bioengineering, Urbana, Illinois, United States; b Washington University School of Medicine in St. Louis, Department of Radiation Oncology, Saint Louis, Missouri, United States

**Keywords:** objective image quality assessment, image restoration, image denoising, deep learning

## Abstract

**Purpose:**

Recently, learning-based denoising methods that incorporate task-relevant information into the training procedure have been developed to enhance the utility of the denoised images. However, this line of research is relatively new and underdeveloped, and some fundamental issues remain unexplored. Our purpose is to yield insights into general issues related to these task-informed methods. This includes understanding the impact of denoising on objective measures of image quality (IQ) when the specified task at inference time is different from that employed for model training, a phenomenon we refer to as “task-shift.”

**Approach:**

A virtual imaging test bed comprising a stylized computational model of a chest X-ray computed tomography imaging system was employed to enable a controlled and tractable study design. A canonical, fully supervised, convolutional neural network–based denoising method was purposely adopted to understand the underlying issues that may be relevant to a variety of applications and more advanced denoising or image reconstruction methods. Signal detection and signal detection-localization tasks under signal-known-statistically with background-known-statistically conditions were considered, and several distinct types of numerical observers were employed to compute estimates of the task performance. Studies were designed to reveal how a task-informed transfer-learning approach can influence the tradeoff between conventional and task-based measures of image quality within the context of the considered tasks. In addition, the impact of task-shift on these image quality measures was assessed.

**Results:**

The results indicated that certain tradeoffs can be achieved such that the resulting AUC value was significantly improved and the degradation of physical IQ measures was statistically insignificant. It was also observed that introducing task-shift degrades the task performance as expected. The degradation was significant when a relatively simple task was considered for network training and observer performance on a more complex one was assessed at inference time.

**Conclusions:**

The presented results indicate that the task-informed training method can improve the observer performance while providing control over the tradeoff between traditional and task-based measures of image quality. The behavior of a task-informed model fine-tuning procedure was demonstrated, and the impact of task-shift on task-based image quality measures was investigated.

## 1 Introduction

The development of image denoising methods for medical imaging applications based on deep neural networks (DNNs) remains an active area of research.^1^^–^^7^ Although learning-based image denoising methods, by conventional design, can improve traditional image quality (IQ) measures such as root mean square error (RMSE) and structural similarity index measure (SSIM), it is well known that such measures may not always correlate with objective task-based IQ measures.^8^^–^^11^ Here and throughout this article, a “task” denotes an image-based inference to be performed by a human or numerical observer. This is because the loss functions that are commonly employed to train such methods do not explicitly take into account the intended task that is to be performed by use of the resulting images. For example, Yu et al.^10^ demonstrated that task-based metrics were not consistent with traditional IQ metrics in a study of DNN-based image denoising related to nuclear medicine imaging. Likewise, Li et al.^9^ reported similar findings and systematically investigated task-related information loss induced by DNN-based denoising methods under different conditions. Task-information loss has also been studied within the context of the learning-based single-image super-resolution problem.^12^

To enhance the utility of an image produced by the use of a learning-based method, information regarding a task can be naturally incorporated into the training procedure.^13^^–^^16^ A variety of task-informed methods employ a hybrid loss comprised of a conventional component and a task-based loss component. For an image reconstruction problem, Adler et al.^13^ proposed such an approach to establish a learned reconstruction operator. Similarly, Ongie et al.^14^ designed a low-dose computed tomography (CT) reconstruction framework to enhance the detectability of signals. For enhancing the utility of denoised images for segmentation tasks, Zhang et al.^17^ proposed a task-informed low-dose CT denoising framework that employed a hybrid loss that incorporated the dice score loss. In a different approach that did not employ the hybrid loss strategy, Han et al.^15^ proposed a perceptual loss-based denoising method.

Although these studies provide valuable insights into the potential of learning-based task-informed image formation and restoration methods, this line of research is relatively new and underdeveloped. Improved utility of the estimated image for the specified task generally comes at the cost of degraded task-agnostic measures of image quality, and understanding this complicated trade-off within the context of a specific problem is important. Although task-related information has been incorporated into loss function designs, the use of transfer learning coupled with constraints on how such information is utilized during model fine-tuning remains unexplored. This is potentially important because previous studies have reported that task-related information may be primarily lost by the deeper layers of a DNN for certain applications.^9^

Another critical issue that is specifically relevant to task-informed learning-based methods for image formation or restoration relates to generalization performance with respect to the task. Tasks in medical imaging applications are generally complicated and can be difficult to comprehensively specify, either analytically or implicitly via the specification of a collection of acquired images. For example, a signal detection task requires the specification of the signal, the background in which it is embedded, and the measurement noise to be detected. All of these quantities are stochastic in nature, and the former two will vary with the subject and disease state in the specified cohort. When a task-informed image formation or restoration method is trained with consideration of a specified detection task, it is anticipated, by design, that the resulting images will possess enhanced utility for performing that particular task. However, at inference time, the characteristics of the signal, background, or noise may differ from those modeled in the original task. This is a phenomenon that we refer to as “task-shift,” indicating that source tasks (used for training) are different from target tasks (used for inference).^18^ Assessing the robustness of a task-informed image formation or restoration method to task-shift is essential to understanding its potential suitability for clinical translation.

In this work, numerical studies are performed to yield insights into fundamental issues related to the incorporation of signal detection task information into a learned image denoising method. Consider that medical images are denoised by use of a DNN and the clinical task of interest is to detect a signal in the denoised images. The following two questions motivate the study design: (1) How do traditional and task-based measures of IQ covary when a conventionally trained DNN is fine-tuned by use of a hybrid task-informed loss function with all weights being frozen except for select deep layers? and (2) What is the impact of task-shift on the IQ measures and what is the relative influence of the source and target task complexity?

A virtual imaging test bed is employed to enable a systematic exploration of these questions. The test bed comprises a stylized computational model of a chest X-ray computed tomography (CT) imaging system coupled with high-fidelity clinical CT images that represent the to-be-imaged objects. From simulated noisy projection data, images that contain lesions are reconstructed. These images are subsequently denoised by use of a learned method. Although a vast number of DNN-based image denoising methods are available and new ones are being developed at a breakneck pace, in this work, a canonical, fully supervised, convolutional neural network (CNN)-based denoising method is purposely adopted. This will facilitate a basic analysis and understanding of the underlying issues that may be relevant to a variety of applications and more advanced denoising or image reconstruction methods. Signal detection and signal detection-localization tasks are considered, and several distinct types of numerical observers are employed to compute estimates of the task performance. The studies will reveal how a task-informed transfer learning approach can influence the tradeoff between conventional and task-based measures of image quality within the context of the considered tasks. In addition, for the first time, insights into the behavior of a learned denoising method when task-shift is present are revealed.

The remainder of the paper is organized as follows. Section 2 describes the necessary background on DNN-based image denoising, signal detection tasks, and numerical observers. The task-informed training method considered in this work is described in Sec. 3. The numerical studies and associated results are described in Secs. 4 and 5, respectively. Finally, the article concludes with a discussion of the key findings in Sec. 6.

## 2 Background

### 2.1 Learning-Based Image Denoising

End-to-end learning-based denoising methods hold significant potential for medical imaging applications.^1^^,^^4^^,^^7^^,^^19^^–^^21^ Given a noisy image $f_{n}\in R^{N}$, where N is the dimension of the image, an end-to-end learning-based denoising method is described generically as

f^=F(fn;Θ), (1)

where F denotes an image-to-image mapping implemented by a DNN that is parameterized by the weight vector Θ and f^∈RN denotes the denoised image. Depending on how the target data are defined when training the DNN, f^ can be interpreted as an estimate of the noiseless image $f\in R^{N}$ or an estimate of a reduced noise version of $f_{n}$. A variety of DNNs have been employed to implement the mapping F,^1^^,^^21^ and convolutional neural networks (CNNs) represent a popular choice.^1^^–^^3^^,^^6^^,^^19^

In addition to the choice of DNN architecture, the specification of the loss function plays a key role in the design of a DNN-based denoising method. Mean square error (MSE) that measures the $L^{2}$ distance between the denoised and target images has been widely employed.^3^^,^^4^^,^^6^^,^^19^^–^^23^ The perceptual loss function has also been used and was reported to be effective in reducing noise while retaining image details,^1^ and the use of adversarial loss functions has been deployed with similar success.^20^^,^^24^ However, such loss functions that are commonly employed in computer vision applications do not explicitly incorporate information regarding a particular medical imaging task. In recent studies, it has been demonstrated that learning-based denoising methods trained by the use of such loss functions can improve traditional IQ measures such as RMSE or SSIM, whereas important information relevant to a downstream detection task is lost.^9^^,^^10^ Such findings motivate the further development and investigation of task-informed learning-based denoising methods.

### 2.2 Formulation of Binary Signal Detection Task

In this study, a binary signal detection task that requires an observer to classify a denoised image f^ as satisfying either a signal-present hypothesis $H_{1}$ or a signal-absent hypothesis $H_{0}$ is considered. These two hypotheses are described as

H0:  f^=F(fb+n), (2a)

H1:  f^=F(fb+s+n), (2b)

where $f_{b+s}\in R^{N}$ and $f_{b}\in R^{N}$ denote signal-present and signal-absent noiseless images, respectively, and $n\in R^{N}$ denotes the measurement noise. A signal-present image $f_{b+s}$ is formulated by inserting a signal image $f_{s}\in R^{N}$ into a background object $f_{b}$. In a signal-known-exactly (SKE) detection task, $f_{s}$ is non-random, whereas, in a signal-known-statistically (SKS) detection task, it is a random process. Similarly, in a background-known-exactly (BKE) detection task, $f_{b}$ is nonrandom, whereas, in a background-known-statistically (BKS) detection task, it is a random process.

In addition, detection-localization tasks in which the signal could be located at one of J distinct locations were considered.^25^ In this case, an observer is required to classify an image as satisfying one of J+1 hypotheses (i.e., one signal-absent hypothesis and J signal-present hypotheses). The imaging processes under these hypotheses are represented as

H0:  f^=F(fb+n), (3a)

Hj:  f^=F(fb+sj+n), (3b)

where j=1,2,…,J and $f_{b+sj}$ is a signal-present noiseless image with the signal at the j’th location.

### 2.3 Numerical Observers for Objective IQ Assessment

In preliminary assessments of medical imaging technologies, numerical observers (NOs) have been employed to quantify task-based measures of IQ for various image-based inferences.^26^ The NOs employed in this study are surveyed below.

#### 2.3.1 Hotelling observer

The Hotelling observer (HO) employs the Hotelling discriminant, which is the population equivalent of the Fisher linear discriminant, and is optimal among all linear observers in the sense that it maximizes the signal-to-noise ratio of the test statistic.^8^^,^^27^ For binary signal detection tasks, the HO test statistic tHO(f^) computed by the use of the denoised data f^ is defined as

tHO(f^)=wHOTf^=(Kf^−1Δf¯)Tf^, (4)

where $w_{\mathrm{HO}}^{T}\in R^{N}$ denotes the Hotelling template, $\Delta \overset{¯}{f}\in R^{N}$ denotes the difference between the ensemble mean of the image data f^ under the two hypotheses $H_{0}$ and $H_{1}$, and Kf^≡12(K0(f^)+K1(f^)). Here, K0(f^)∈RN×N and K1(f^)∈RN×N denote the covariance matrices corresponding to f^ under $H_{0}$ and $H_{1}$, respectively.

In some cases, the covariance matrices K0(f^) and K1(f^) are ill-conditioned, and therefore, their inverse cannot be stably computed. To address this, a regularized HO (RHO) can be employed that implements the test statistic tRHO(f^) as^9^

tRHO(f^)=wRHOTf^=(Kα+Δf¯)Tf^, (5)

where $K_{\alpha }$ represents a low-rank approximation of Kf^ that is formed by keeping only the singular values of Kf^ greater than $\alpha \sigma_{\max}$. Here, α is a tunable parameter, and $\sigma_{\max}$ represents the largest singular value of Kf^. Finally, $K_{\alpha }^{+}$ is the Moore–Penrose inverse of $K_{\alpha }$.

#### 2.3.2 Channelized Hotelling observer

A channelized HO (CHO) is formed when the HO is employed with a channeling mechanism. When implemented with anthropomorphic channels and an internal noise mechanism, the CHO can be interpreted as an anthropomorphic observer and attempts to predict the human observer performance.^28^^,^^29^ In addition, the channeling mechanism can be employed to reduce the dimensionality of the image data when the image data are insufficient to accurately estimate the covariance matrix. Let T denote a channel matrix and v≡Tf^ denote the corresponding channelized image data. The CHO test statistic tCHO(f^) is given as

tCHO(f^)=[(Kv+Kint)−1Δv¯]T(v+vint), (6)

where $K_{v}$ denotes the covariance matrix of the channelized data v, $K_{\mathrm{int}}$ denotes the covariance matrix of the channel internal noise, and $v_{\mathrm{int}}$ is a noise vector sampled from a Gaussian distribution $N({0,K}_{\mathrm{int}})$. Based on previous studies,^29^ in this work, $K_{\mathrm{int}}$ is defined as

$$K_{\mathrm{int}}=\epsilon \cdot \mathrm{diag}(K_{v}),$$ (7)

where $\mathrm{diag}(K_{v})$ represents a diagonal matrix with diagonal elements from $K_{v}$ and ϵ is the internal noise level. The parameters of the difference-of-Gaussian (DOG) channels and the internal noise level employed in this study are described in Sec. 4.3.4.

#### 2.3.3 Learned NOs

Recently, several machine learning methods have been proposed to establish NOs.^30^^–^^36^ The single-layer neural network (SLNN)-based NO (SLNN-NO) is a special learned NO that has the shallowest architecture, possessing only a single fully connected layer with a bias term and a sigmoid activation function. This architecture can be employed for different tasks through the specification of the loss function. For example, the binary cross entropy (BCE) can be used to train the SLNN-NO for binary signal detection tasks, whereas the categorical cross-entropy loss function can be employed for detection-localization tasks. A SLNN-based method has also been proposed to approximate the HO.^30^ This NO will be referred to as the SLNN-HO and will also be employed in the studies below. The SLNN-HO is useful when the estimation and/or inversion of the image covariance matrix is intractable.

## 3 Task-Informed Training Method

A transfer learning approach is investigated in which a DNN is pre-trained by the use of a conventional (non-task-informed) loss function $L_{p}$ and subsequently fine-tuned by the use of a hybrid loss $L_{\text{Hybrid}}$ that includes a task component $L_{t}$. The fine-tuning of the denoising network is constrained to the last several layers instead of re-training the whole network. This is motivated by a recent study by Li et al.^9^ who demonstrated that, at least for linear CNN-based denoising networks, the degradation of task-relevant information primarily occurs in the last layers. This behavior can be explained by noting that the last layer of the denoising network transforms a high-dimensional feature tensor into the denoised output image. Therefore, the transform possesses a null space and is non-invertible.^9^ A hybrid loss function $L_{\text{Hybrid}}$ is defined as^13^

$$L_{\text{Hybrid}}(\Theta_{1},\Theta_{o})=(1-\lambda )\cdot L_{p}(\Theta_{1})+\lambda \cdot L_{t}(\Theta_{1},\Theta_{o}),$$ (8)

where λ∈[0,1] is a scalar parameter, $L_{p}$ is the physical loss component, $L_{t}$ is the task component, $\Theta_{1}$ is the vector of weight parameters associated with the trainable layers in the pretrained denoising network, and $\Theta_{o}$ denotes the vector of weight parameters of the neural network (NN)-based NO used to compute the task component $L_{t}$. The task component is designed to measure the performance of a NO on a specific task. By appending a network-based NO to the pretrained denoising network, the denoised image can be transformed into a scalar that is used to compute the task-specific component. The trainable layers in the pretrained denoising network are jointly trained with the NO. By employing this training strategy, the NO used to compute $L_{t}$ can be easily adapted to different tasks. The details of the proposed task-informed training method are described below and summarized in Procedure 1.

**Procedure 1:** General procedure of the task-informed training method

**Input:**
• Θ: Weight parameters of the denoising network F with N layers;
• $\Theta_{1}$: Weight parameters of the last $N_{\text{train}}$ trainable layers of F;
• $\Theta_{2}$: Weight parameters of the first $\left(N-N_{\text{train}}\right)$ layers of the denoising network F, $\Theta =\{\Theta_{1},\Theta_{2}\}$;
• $\Theta_{o}$: Weight parameters of an appended observer used to compute the task component $L_{t}$;
• $L_{p}$: Physical loss for pre-training and task-informed training F;
• $L_{t}$: Task-based loss for task-informed training F;
• $L_{\text{Hybrid}}$: The hybrid loss formulated by $L_{p}$ and $L_{t}$ and weighted by the parameter λ defined in Eq. (8);
• $D_{1}$: Dataset for pre-training F;
• $D_{2}$: Dataset for task-informed training F;
**Procedure:**
1. Pretrain the denoising network F and optimize weight parameters Θ by use of $D_{1}$ and physical loss function $L_{p}$;
2. Append the observer with initial weight parameters $\Theta_{o}$ to the pretrained F;
3. Set the weight parameters $\Theta_{1}$ that correspond to the last $N_{\mathrm{train}}$ convolutional layers of the pretrained F to be trainable;
//The other trained weight parameters $\Theta_{2}$ are fixed;
4. Given initial setting of $\Theta_{o}$ and pre-trained Θ, jointly tune the weight parameters $\Theta_{1}$ and train $\Theta_{o}$ by use of $D_{2}$ and the hybrid loss function $L_{\text{Hybrid}}$;
5. Output the task-informed denoising network F with optimized weight parameters Θ.
**Output:** The denoising network F with optimized weight parameters Θ after task-informed training by use of $L_{\text{Hybrid}}$.

Mean squared error (MSE) and mean absolute error are commonly employed choices for $L_{p}$. The selection of the task-based loss component is based on specific tasks. In this paper, binary signal detection tasks were considered, and the specific formulation of $L_{\mathrm{Hybrid}}$ is described in Sec. 4.2.

## 4 Numerical Studies

Computer-simulation studies using a stylized X-ray CT virtual imaging test bed were conducted to gain insights into fundamental issues described in Sec. 1. Signal-known-statistically (SKS) with background-known-statistically (BKS) signal detection and signal detection-localization tasks were considered. Both the SLNN-NO and SLNN-HO were employed to compute estimates of the task performance, which was employed to evaluate the impact of a task-informed training procedure on the considered denoising network described in Procedure 1.

### 4.1 Virtual Imaging Pipeline

The Lung Image Database Consortium image collection^37^ was employed to generate signal-present (SP) images $f_{b+s}$ and signal-absent (SA) images $f_{b}$ to perform binary signal detection tasks as defined in Eq. (2). This database consists of 243,945 2D image slices from 1018 3D thoracic CT reconstructed images, in which 10,706 image slices contain annotated nodules. A total of 100,000 SA images were formed by extracting regions of interest (ROIs) of a dimension of 120×120  pixels from normal lung areas from several central slices. To generate SP images, an established insertion method^38^ was employed to insert realistic nodules into 50,000 generated SA images. In SP images, the centroids of the nodules were either located at a fixed location or at random locations subject to the specific tasks described in Sec. 4.3. The generated SP and SA images were utilized as the target (normal-dose) CT images f.

The corresponding noise-enhanced (low-dose) images $f_{n}$ were generated by degrading the target images f described above. A canonical fan-beam CT imager with a linear detector geometry was considered for noise simulation. To produce these images, the true continuous-to-discrete forward operator was approximated by a discrete-to-discrete operator H that was implemented by use of the Radon-torch toolbox.^39^ The scanning angular range of the modeled fan-beam system was 360 degrees, and 256 evenly spaced tomographic views were considered. The assumed distance between the X-ray source and the center of the object and the distance between the detector and the center of the object were 400 and 400 mm, respectively. The number of detector elements was 512, and each element was 0.8 mm in size. During the simulated imaging process, the forward operator was applied to the entire chest cross-section, covering the system’s full field of view.

**Fig. 1:**
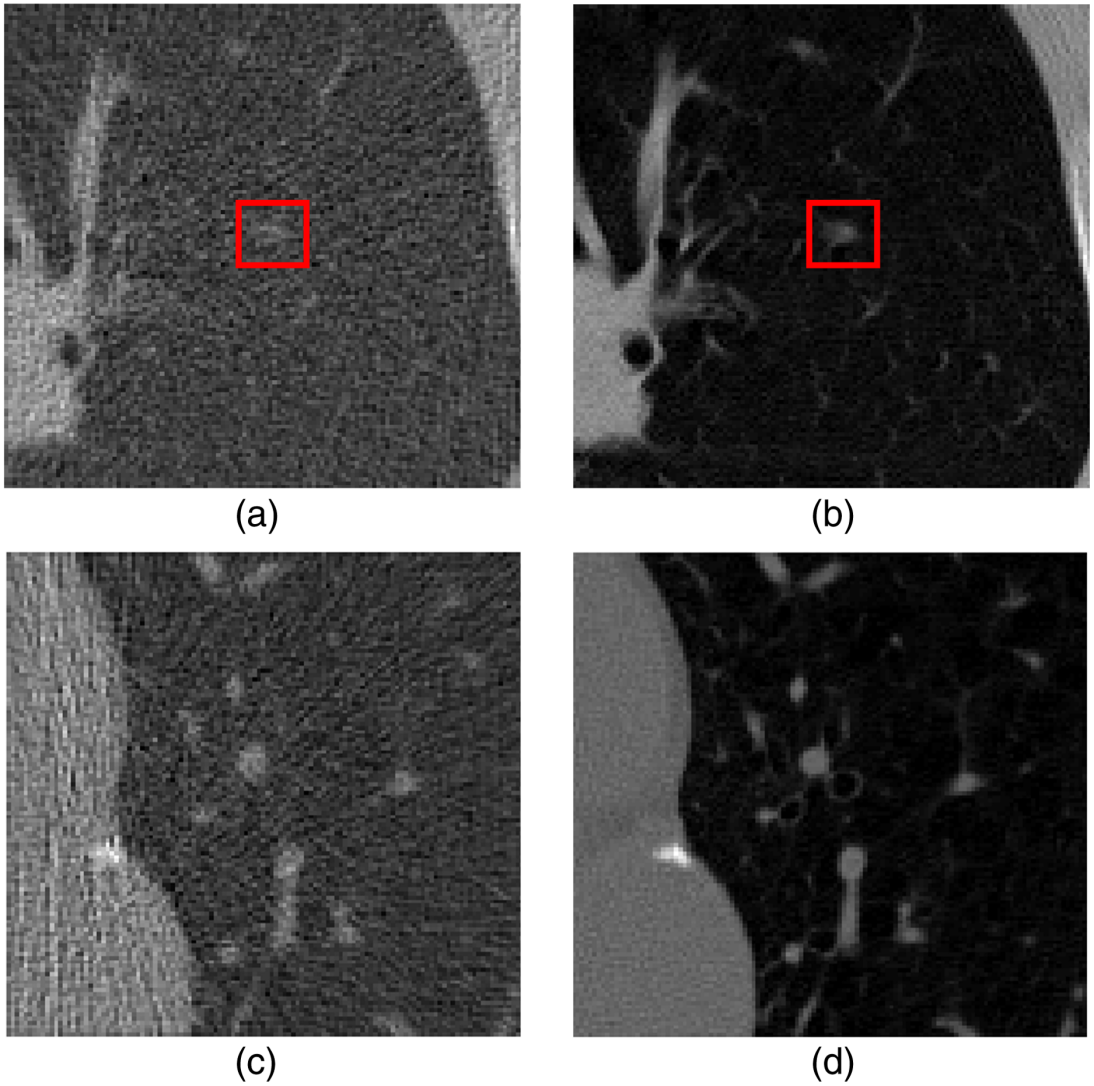
Examples of (a) noisy (low-dose) signal-present ROI, (b) target (normal-dose) signal-present ROI, (c) noisy (low-dose) signal-absent ROI, and (d) target (normal-dose) signal-absent ROI, which were extracted from reconstructed cross-section images. The red box contains the signal.

Noise-enhanced projection data g were generated as^8^^,^^39^

$$g=T^{-1}(\text{Poi}(T(\mathrm{Hf}))),$$ (9)

where Poi(·) is a Poisson noise generator acting on the transformed measurement data T(Hf). Here, $T(x)=I_{0}\;\exp(-x)$, and $T^{-1}(x)=\log(\frac{I_{0}}{x})$, where $I_{0}$ is the beam intensity. The noisy (low-dose) images $f_{n}$ were then reconstructed from g by the use of a filtered back-projection reconstruction algorithm that employed a Ram-Lak filter.^40^ As described below, the proposed denoising method was applied to regions of interests (ROIs) of dimension 120×120  pixels within the reconstructed images. These ROIs were situated within the lung area, with their center locations uniformly distributed over that region. Figure 1 shows examples of ROIs employed in our studies.

### 4.2 Training and Validation Details

#### 4.2.1 Architecture and loss function for denoising networks

**Fig. 2:**
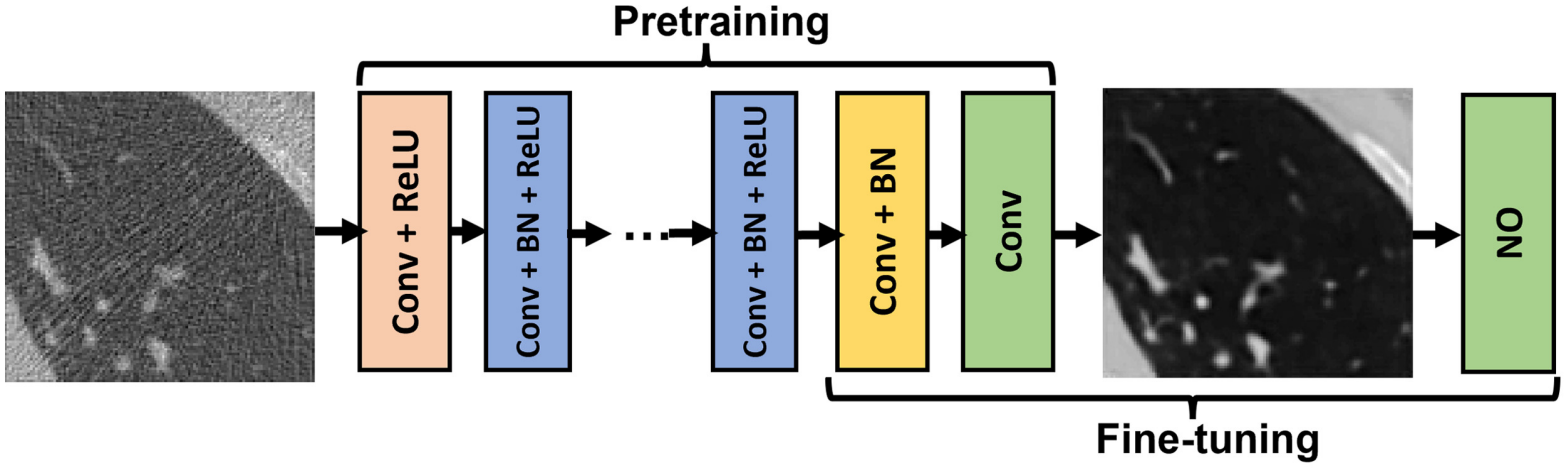
CNN-based denoising network investigated in this study.

The canonical CNN architecture of depth D depicted in Fig. 2 was employed with the task-informed training method to establish an end-to-end learned denoising method. It is important to note that the assessment studies described below can be readily repeated with any other DNNs. The network input was a reconstructed noisy image $f_{n}$ of dimension 120×120, and the output was a denoised image f^ with the same dimensions. The CNN contained four types of layers. The first layer was a Conv+ReLU layer, in which 64 convolution filters of dimension 3×3×1 were applied to generate 64 feature maps. In each of the 2nd to $(D-2{)}^{\mathrm{th}}$ Conv + BN + ReLU layers, 64 convolution filters of dimension 3×3×64 were employed, and batch normalization was included between the convolution and ReLU operations. In the $(D-1{)}^{\mathrm{th}}$ Conv + BN layer, 64 convolution filters of dimension 3×3×64 were employed, and batch normalization was performed. In the last Conv layer, one single convolution filter of dimension 3×3×64 was employed to form the final denoised image of dimension 120×120.

Let $f^{\left(j\right)}$ denote a given SA or SP target (normal-dose) image, and let $f_{n}^{\left(j\right)}$ denote the corresponding noise-enhanced (low-dose) image. Given a collection of J paired training data $\{(f_{n}^{\left(j\right)},f^{\left(j\right)}){\}}_{j=1}^{J}$, the denoising network was pretrained by minimizing the MSE loss function:

$$L_{\mathrm{MSE}}(\Theta )=\frac{1}{J}\overset{J}{\underset{j=1}{\sum }}{\left\|F(f_{n}^{\left(j\right)};\Theta )-f^{\left(j\right)}\right\|}_{2}^{2},$$ (10)

where the vector Θ denotes the weight parameters of the denoising network.

#### 4.2.2 Architecture and loss function for the NN-based observers used to compute $L_{t}$

The physical loss function $L_{p}$ in Eq. (8) was defined by an MSE loss. The task component $L_{t}$ of the hybrid loss function was computed by the use of either the SLNN-NO or SLNN-HO, as described next.

The SLNN-NO consisted of a fully connected layer along with a sigmoid activation function. The BCE loss function was employed to train the SLNN-NO. Let $\{(f_{n}^{\left(j\right)},y^{\left(j\right)}){\}}_{j=1}^{J}$ denote the image data $f_{n}^{\left(j\right)}$ and the corresponding label $y_{j}\in \{0,1\}$. The BCE loss function $L_{\mathrm{BCE}}(\Theta_{1},\Theta_{o})$ is expressed as^30^

$$L_{\mathrm{BCE}}(\Theta_{1},\Theta_{o})=-\overset{J}{\underset{j=1}{\sum }}\log\;p(y_{j}|f_{n}^{\left(j\right)},\Theta_{1},\Theta_{o}).$$ (11)

Here, $\Theta_{1}$ is the vector of weight parameters associated with the trainable layers in the pretrained denoising network, and the vector $\Theta_{o}$ denotes the weight parameters of the fully connected layer of the appended SLNN-NO.

Differently, the SLNN-HO loss function $L_{\mathrm{HO}}(\Theta_{1},\Theta_{o})$ is expressed as^30^

$$L_{\mathrm{HO}}(\Theta_{1},\Theta_{o})=\frac{1}{J}\overset{J}{\underset{j=1}{\sum }}\{(1-y_{j})[\Theta_{o}^{T}(F(f_{n}^{\left(j\right)};\Theta_{1})-{\overset{¯}{f}}_{0}){]}^{2}\;+y_{j}[\Theta_{o}^{T}(F(f_{n}^{\left(j\right)};\Theta_{1})-{\overset{¯}{f}}_{1}){]}^{2}\}-2\Theta_{o}^{T}\Delta \overset{¯}{f},$$ (12)

where ${\overset{¯}{f}}_{0}=\frac{2}{J}\overset{J}{\underset{j=1}{\sum }}(1-y_{j})F(f_{n}^{\left(j\right)};\Theta_{1})$, ${\overset{¯}{f}}_{1}=\frac{2}{J}\overset{J}{\underset{j=1}{\sum }}y_{j}F(f_{n}^{\left(j\right)};\Theta_{1})$, and $\Delta \overset{¯}{f}={\overset{¯}{f}}_{1}-{\overset{¯}{f}}_{0}$.

#### 4.2.3 Datasets and denoising network training details

The standard convention of utilizing separate training/validation/testing datasets was adopted. The training dataset included 40,000 pairs of noisy signal-present and signal-absent images along with the corresponding target (normal noise) images. The validation dataset including 200 signal-present images and 200 signal-absent images, and the corresponding target (normal noise) images was randomly selected from the training dataset. Finally, the testing dataset comprised 10,000 signal-present images and 10,000 signal-absent noisy images. For task-informed model training with the hybrid loss function $L_{\text{Hybrid}}$, the same training dataset described above was employed to fine-tune the denoising network. The validation and testing datasets used for pretraining were also employed to evaluate the performance of the fine-tuned denoising networks.

In both the pretraining and task-informed fine-tuning stages, the denoising networks were trained on mini-batches at each iteration by the use of the Adam optimizer^41^ with a learning rate of 0.0001. Each mini-batch contained 50 signal-present images and 50 signal-absent images that were randomly selected from the training dataset. The network model that possessed the best performance on the validation dataset was selected for use. The Keras library^42^ was employed for implementing and training all networks on a single NVIDIA TITAN X GPU.

### 4.3 Objective Evaluation of Image Quality

#### 4.3.1 SKS/BKS binary signal detection tasks with fixed signal locations

The task-informed training method was evaluated for SKS/BKS binary signal detection tasks for which known signal locations were considered. The centroids of the nodules were located at the center of the extracted ROIs. The incident flux $I_{0}=e^{11}$ was used to determine the noise level in the simulated noisy images.

When fine-tuning the denoising networks, the SLNN-NO and SLNN-HO were employed as NOs to compute the task component $L_{t}$ in Eq. (8). Here, $L_{t}$ was defined as the BCE and HO loss functions in Sec. 4.2.2 when SLNN-NO and SLNN-HO were employed, respectively. The SLNN-NO, HO, RHO, and DOG-CHO were employed for subsequent assessments of image quality. It should be noted that, for the case in which the SLNN-NO was employed to compute $L_{t}$, the SLNN-NO employed for objective image quality assessment was trained on the denoised estimates, and it was not identical to that used to compute $L_{t}$. The use of these NOs for evaluation represented a situation in which the NO for evaluation may not be identical to the NO used to optimize the denoising networks. The weight parameters λ={0.01,0.1,0.3,0.5,0.7,0.9,0.99} in Eq. (8) were considered. Only the last three convolutional layers of the denoising network were set to be trainable for both cases. Based on these settings, the impact of the weight parameter λ on the performance of the considered NOs was investigated.

#### 4.3.2 SKS/BKS binary signal detection tasks with random signal locations

In this case, the centroids of the nodules were randomly located within the lung area of extracted ROIs by the use of a uniform probability density function. The incident flux $I_{0}=e^{11}$ was used to determine the noise level of the simulated low-dose images. The SLNN-NO was used to compute task component $L_{t}$ in Eq. (8), considering that the SLNN-NO can be employed when the signal is randomly located. The trained SLNN-NO was subsequently utilized to evaluate the performance of fine-tuned denoising networks. This represented a situation in which the same observer was used for both training and evaluation.

To assess the impact of the weight parameter λ on the performance of the SLNN-NO, the weight parameters λ={0.01,0.1,0.3,0.5,0.7,0.9,0.99} in Eq. (8) were considered. The number of trainable layers was also swept from 0 to 4.

#### 4.3.3 Investigation of the impact of task-shifts

##### Test cases with different weight parameters λ

A study was designed to investigate the robustness of the task-informed image denoising method to task-shift. First, binary signal detection tasks with fixed signal locations were considered for model training (source tasks), whereas tasks with random signal locations were considered for evaluation (target tasks). Next, the tasks with random signal locations were used as source tasks, and the tasks with fixed locations were considered target tasks. Detection-localization tasks were also considered. The tasks with two and four possible signal locations were considered to be both source/target and target/source tasks to study the impact of task-shift. The considered test cases are outlined in Table 1.

**Table 1:** Test cases designed for the investigation of the impact of task-shifts described in Sec. 4.3.3. Here, BSD and D&L represent binary signal detection tasks and detection-localization tasks, respectively.

Test case	Source task	Target task
**#1**	BSD with fixed signal locations	BSD with random signal locations
**#2**	BSD with random signal locations	BSD with fixed signal locations
**#3**	D&L with two signal locations	D&L with four signal locations
**#4**	D&L with four signal locations	D&L with two signal locations

The SLNN-NO was employed to compute task component $L_{t}$ in Eq. (8), and only the last three convolutional layers were set to be trainable. For evaluations, SLNN-NOs were independently trained on training datasets for target tasks. The SLNN-NO performance under the situations without task-shift was considered the reference. The weight parameters λ={0.01,0.1,0.3,0.5,0.7,0.9,0.99} in Eq. (8) were considered to investigate the impact of task-shifts when the weight of the task-based component varies.

##### Test cases with gradually increased mismatches in source/target tasks

Studies were designed to simulate situations in which the mismatch in the source task and target task was gradually increased. In the case of a binary signal detection task, the source task was a binary signal detection task with a fixed signal location. For the target tasks, the signal was randomly located within circles with radii r={4,8,12,16,20}. Detection-localization tasks were also considered. Here, the source task possessed two fixed signal locations, and tasks with {2,3,4,5,6} fixed signal locations were considered target tasks. In these tasks, the signal was randomly located within one of the considered possible locations.

The SLNN-NO was employed to compute the task component $L_{t}$ in Eq. (8) for network training. Only the last three convolutional layers were set to be trainable, and the weight parameter λ=0.7. For evaluations, SLNN-NOs were independently trained on different training datasets designed for target tasks. The SLNN-NO performance under the situations without task-shift was considered a reference.

#### 4.3.4 Numerical observer computation

Both the HO and RHO were employed for objective image quality assessment because they are optimal linear observers. For computing the HO and RHO test statistics, the covariance matrix Kf^ was empirically estimated by the use of 40,000 signal-present and 40,000 signal-absent images. When computing the RHO test statistic, the threshold parameter α in Eq. (5) was swept from 1e−1 to 1e−7, and the corresponding detection performance was estimated based on a separate validation dataset, including 200 signal-present images and 200 signal-absent images. The value that led to the best RHO detection performance was selected.

For computing the CHO test statistic, 2000 signal-present and 2000 signal-absent images were utilized to empirically estimate the channelized covariance matrix. A set of 10 DOG channels^29^ was employed with channel parameters $\sigma_{0}=0.005$, α=1.4, and Q=1.67. The internal noise level ϵ was 2.5, which was the same value employed by Abbey and Barrett.^29^

To independently train the SLNN-NO for objective image quality assessment, 40,000 signal-present images and 40,000 signal-absent images were employed. These learned-NOs were trained by use of the Adam optimizer^41^ with a learning rate of 0.0001.

#### 4.3.5 Evaluation metrics

Both traditional and task-based measures of IQ were employed for assessments. Receiver operating characteristic (ROC) analysis was conducted, and area under the curve (AUC) values were computed and employed as a figure-of-merit for task-based measures. The ROC curves were fit by the use of the Metz-ROC software^43^ that employs the proper binormal model.^44^ The uncertainty of the AUC values was estimated as well. Two commonly used traditional metrics (i.e., RMSE and SSIM) were employed as task-agnostic measures to assess the denoised images.

## 5 Results

In Secs. 5.1 and 5.2, the results are presented to reveal the impact of the task-informed training method on the tradeoff between conventional and objective measures of image quality within the context of the considered tasks. In addition, in Sec. 5.3, the results are reported to investigate the impact of denoising on objective measures of IQ when task-shift was introduced.

### 5.1 Results for the Case with Fixed Signal Locations

The task-informed training method was evaluated for SKS/BKS binary signal detection tasks in which known signal locations were considered. Here, these tasks were employed for both training and evaluation of the task-informed training method in which no task-shift was present. Section 5.1.1 describes the impact of the weight parameter λ on the performance of the employed NOs. In Sec. 5.1.2, the changes in the covariance matrix induced by the task-informed training method were also investigated to gain insights into the observer performance.

#### 5.1.1 Impact of the weight parameter λ

**Fig. 3:**
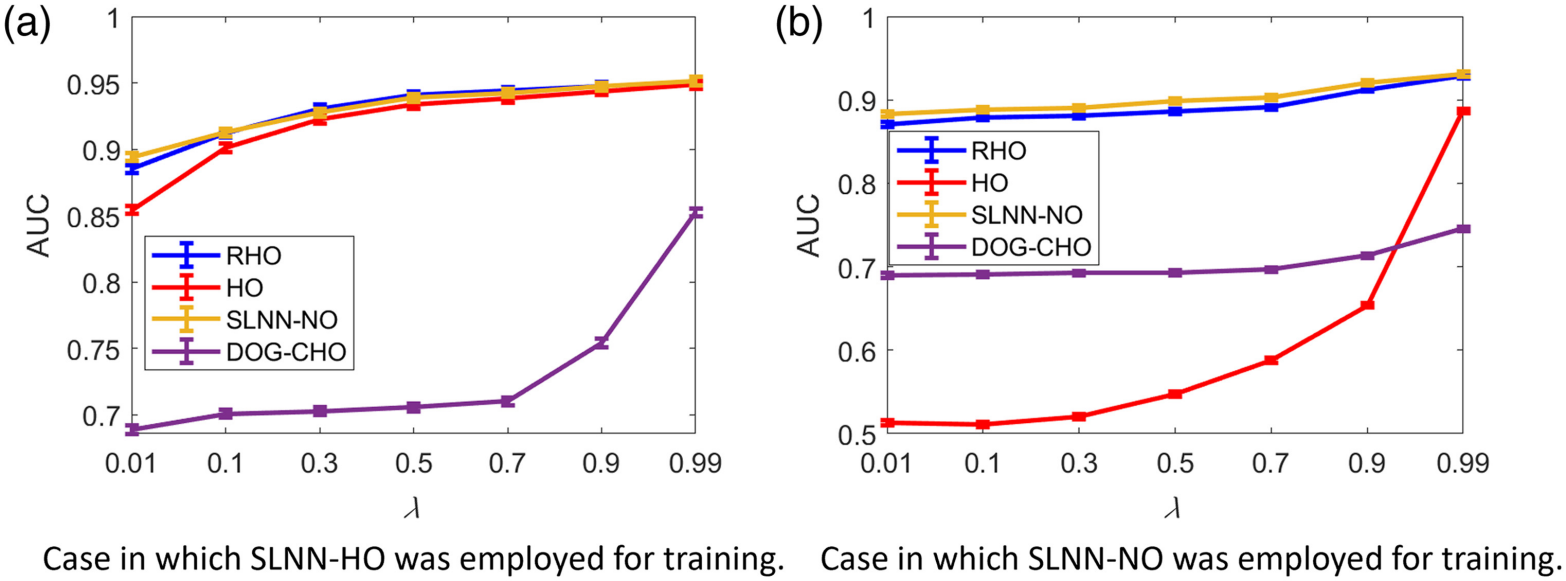
Relationships between AUC and the weight parameter λ in the hybrid loss $L_{\text{Hybrid}}$ when different NOs were employed for objective image quality assessment. Panels (a) and (b) the case in which the SLNN-HO and SLNN-NO were employed to compute the task component $L_{t}$ in Eq. (8), respectively, during model training. For both cases, the performance of the four different NOs increased as a function of λ, confirming that the task-informed denoising method can improve the NO performance even when different NOs were used for model training and for image quality assessment.

The impact of the weight parameter λ in Eq. (8) on the signal detection performance as measured by AUC is shown in Fig. 3. Both the SLNN-NO and SLNN-HO described in Sec. 4.2.2 were considered to be the NO to compute task component $L_{t}$ in Eq. (8). The signal detection performance was evaluated by the use of the SLNN-NO, HO, RHO, and DOG-CHO acting on the denoised images. For both cases, the performance of the four different NOs on the denoised images was higher when larger λ values (larger weight for task-based loss) were considered. Those results confirm that the task-informed training method can improve the NO performance even when the NOs employed for objective image quality assessment were different from the NO used to compute $L_{t}$ during model training.

Figure 3 yields two additional noteworthy findings. First, for the case in which the SLNN-HO was employed for training [panel (a)], the performance of the HO employed for objective image quality assessment was relatively high (statistically equivalent to that of the SLNN-NO and RHO). However, this relatively high HO performance was only observed for very large λ values (e.g., 0.99) in which SLNN-NO [panel (b)] was employed. The HO performance was much lower when relatively small λ values (i.e., 0.01-0.9) were employed. The RHO performance was employed as a reference and was relatively high for all cases. These observations suggest that, for the case in which SLNN-HO was employed for training, the second- and potentially higher-order statistical properties of the images were optimized to benefit the HO performance, but such behavior did not occur in the case in which SLNN-NO with small λ values was considered. Second, when SLNN-HO was used for training, the performance of DOG-CHO was greatly improved for large λ values and was not significantly improved for other cases. This observation indicates that the DOG channels were “closer” to efficient channels when λ was appropriately selected. However, this behavior was not observed in the case in which SLNN-NO was employed for training.

#### 5.1.2 Changes in covariance matrix induced by the task-informed training method

**Fig. 4:**
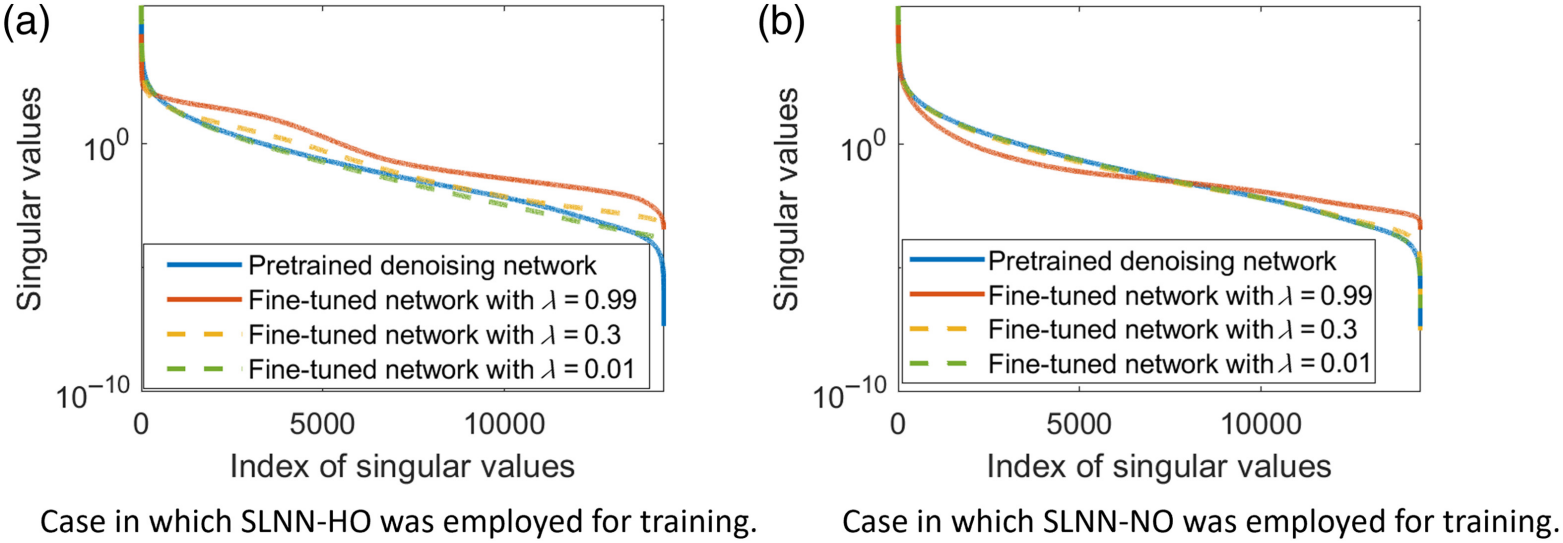
Singular value spectra of covariance matrices corresponding to the denoised images. The related task-informed denoising methods were trained using the hybrid loss $L_{\text{Hybrid}}$ with different λ. Panels (a) and (b) the case in which the SLNN-HO and SLNN-NO were employed to compute the task component $L_{t}$ in Eq. (8), respectively, during model training. These results demonstrate that the task-informed denoising method could mitigate the ill-conditioning of the covariance matrices and possesses the potential to improve the image statistics that are important for signal detection. The results of this analysis were consistent with the previously discussed results shown in Fig. 3.

To gain insights into the behavior of the HO performance, the singular value spectra of the covariance matrices corresponding to the images denoised by task-informed training method were further examined. The results, shown in Fig. 4, reveal that the covariance matrix corresponding to the denoised images produced by the use of the pretrained denoising network was ill-conditioned, whereas that corresponding to the denoised images produced by the use of the fine-tuned denoising network was well-conditioned when SLNN-HO was employed to compute the task component $L_{t}$ in Eq. (8). However, for the case in which SLNN-NO was employed to compute $L_{t}$, a similar observation only occurred for very large λ values (e.g., 0.99) in Eq. (8), and the covariance matrices were still ill-conditioned for small λ values [Fig. 4(b)]. The results of this analysis were consistent with the previously discussed results shown in Fig. 3 and indicated that the task-informed training method may improve the image statistics that are important for signal detection.

### 5.2 Results for the Case with Random Signal Locations

The impact of the task-informed training method on the tradeoff between conventional and objective measures of IQ was investigated by considering SKS/BKS binary signal detection tasks with random signal locations for both training and evaluation. Section 5.2.1 describes the impact of the weight parameter λ and the number of trainable layers on the SLNN-NO performance. In Sec. 5.2.2, a study was also performed to investigate whether the loss of task-relevant information primarily occurs in the last several layers when the denoising network depths increase.

#### 5.2.1 Impact of the weight parameter λ and number of trainable layers

**Fig. 5:**
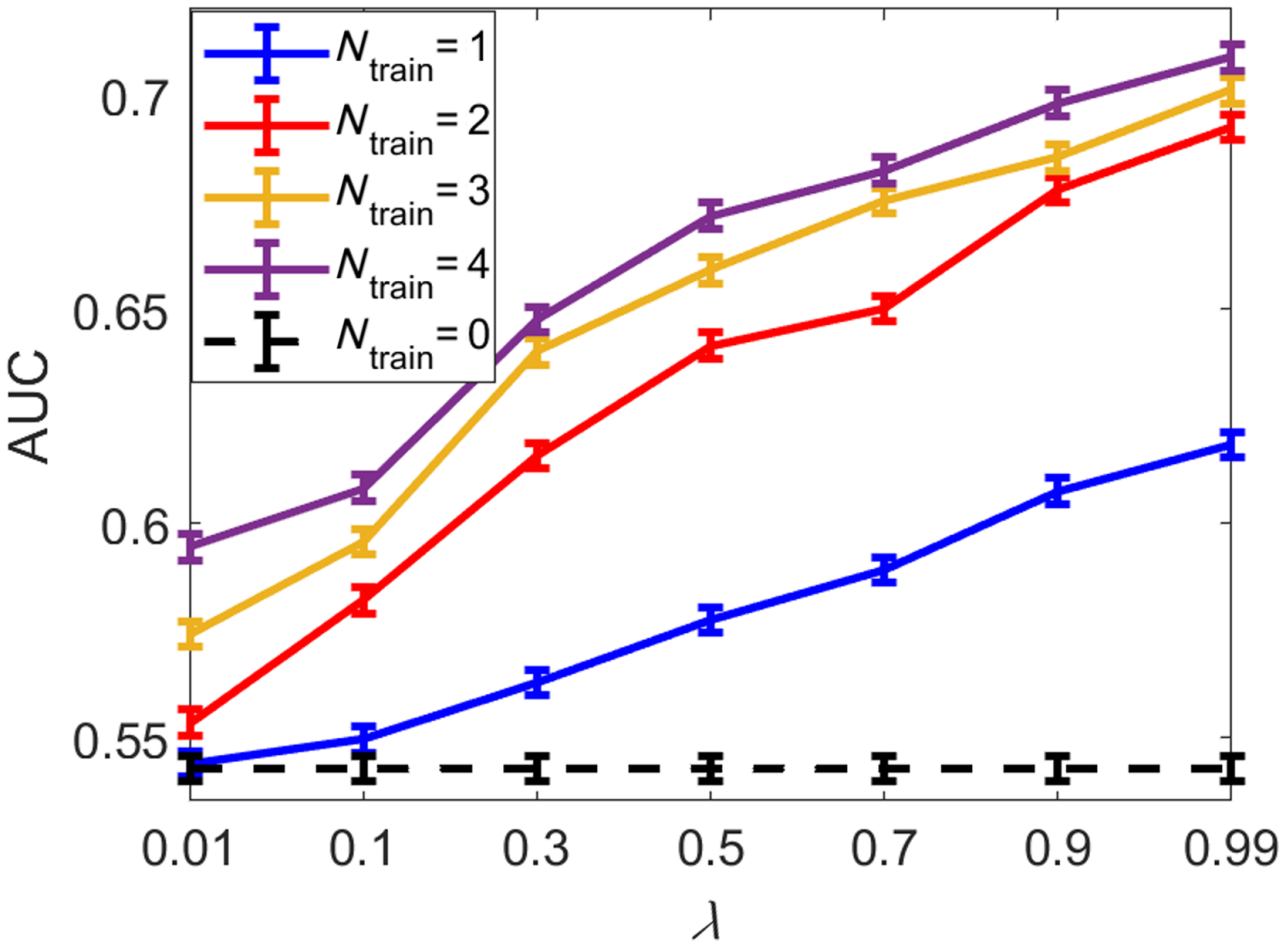
Relationships between AUC and the weight parameter λ in Eq. (8) when different numbers of trainable layers ($N_{\text{train}}$) in the denoising network were considered. The SLNN-NO was employed for both denoising model training and objective image quality assessment. The dashed line at the bottom represents the SLNN-NO performance on images produced by the pre-trained, non-task-informed, denoising network.

**Fig. 6:**
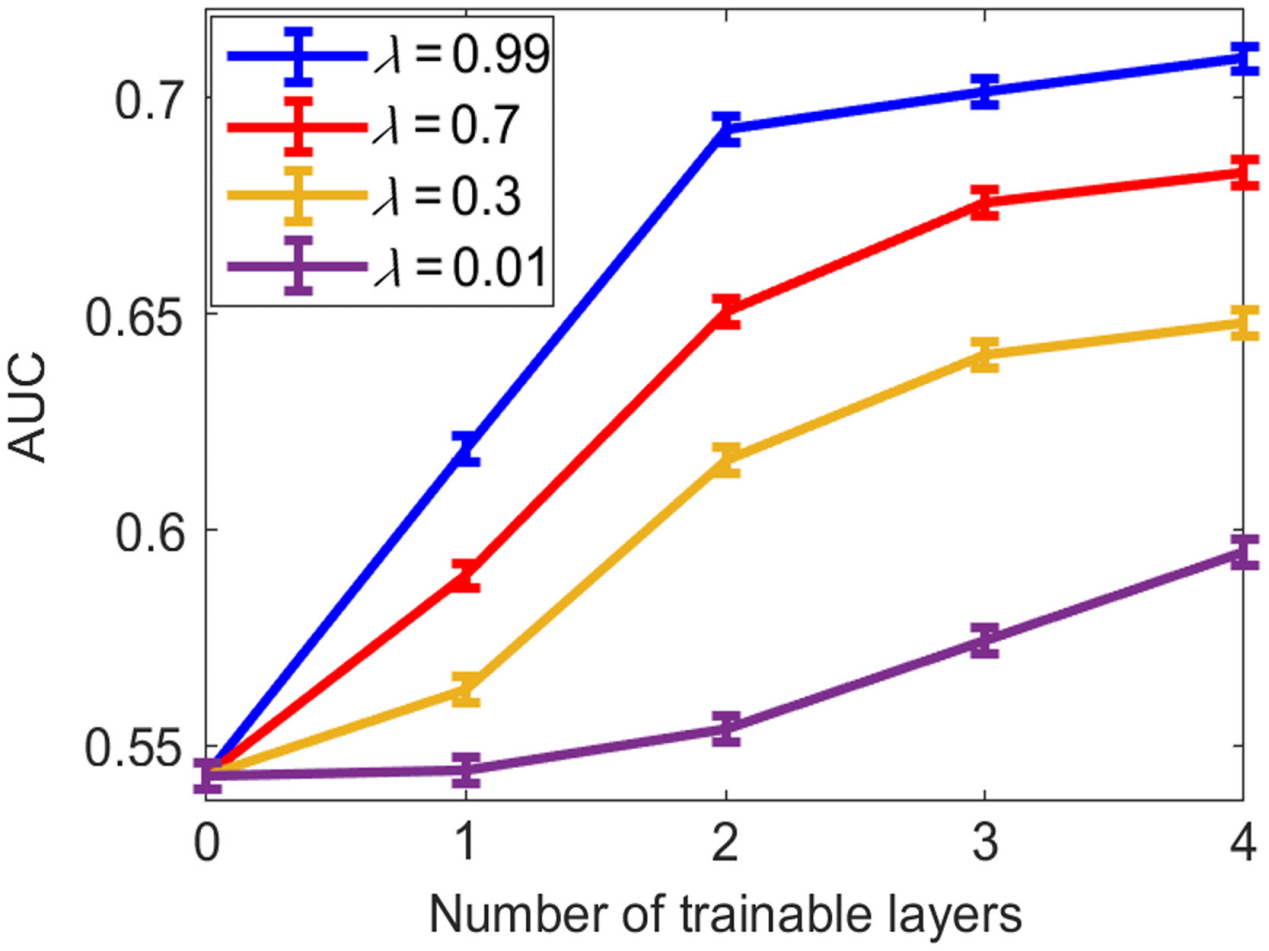
Relationships between AUC and the number of trainable layers in the denoising network when the task component $L_{t}$ in $L_{\text{Hybrid}}$ was weighted with different λ. The SLNN-NO was employed for both denoising model training and objective image quality assessment. Significant improvements in task performance were achieved by fine-tuning the last several convolutional layers (e.g., 0 to 2), whereas improvement was less significant when more layers were subject to refinement.

The impacts of the weight parameter λ in Eq. (8) and the number of trainable layers on the signal detection performance as measured by the SLNN-NO are shown in Figs. 5 and 6, respectively. Here, the SLNN-NO employed to compute the task component $L_{t}$ in Eq. (8) was also employed to assess the signal detection performance. For comparison, the impact on traditional measures of IQ is demonstrated in Table 2. It was observed that, after the task-informed model training, the SLNN-NO signal detection performance was improved, whereas the traditional measures of IQ were degraded compared with those achieved by the pre-trained denoising network.

**Table 2:** Relationships between RMSE and the number of trainable layers in the denoising network and the weight parameter λ in $L_{\text{Hybrid}}$. The quantity $N_{\text{train}}$ denotes the number of trainable layers. The values shown in the column related to $N_{\text{train}}=0$ represent cases in which RMSE and SSIM were calculated on images denoised by the pretrained non-task-informed denoising network. Additional details are provided in Sec. 5.2.1.

$N_{\text{train}}$	λ	0	1	2	3	4
RMSE	0.01	1.3437	1.3498	1.3485	1.3481	1.3477
	0.1	1.3437	1.3503	1.3499	1.3498	1.3495
	0.3	1.3437	1.3507	1.3506	1.3501	1.3498
	0.5	1.3437	1.3506	1.3514	1.3534	1.3546
	0.7	1.3437	1.3509	1.3549	1.3613	1.3667
	0.9	1.3437	1.3775	1.3988	1.4147	1.4244
	0.99	1.3437	1.7178	1.9304	2.1127	2.2595
SSIM	0.01	0.9416	0.9408	0.9409	0.9410	0.9414
	0.1	0.9416	0.9407	0.9409	0.9411	0.9412
	0.3	0.9416	0.9407	0.9408	0.9409	0.9409
	0.5	0.9416	0.9405	0.9407	0.9407	0.9408
	0.7	0.9416	0.9404	0.9401	0.9394	0.9391
	0.9	0.9416	0.9391	0.9381	0.9369	0.9368
	0.99	0.9416	0.9197	0.9024	0.8755	0.8738

As shown in Fig. 5 and Table 2, for all numbers of trainable layers, the task performance increased as a function of λ. In addition, the degradation of traditional metrics was significant for relatively large λ but insignificant for small λ values (i.e., λ=0.01−0.7). As expected, the tradeoff between traditional and task-based measures of IQ can be controlled by λ. For example, when λ=0.7, the resulting AUC value was greatly improved to that of λ=0.01, whereas the RMSE and SSIM were statistically equivalent to that of λ=0.01.

As shown in Fig. 6, significant improvements in the task performance were achieved by fine-tuning the last (or last several) convolutional layer(s) (e.g., 0 to 2), whereas improvement was insignificant when more layers were trainable. For traditional IQ metrics (i.e., RMSE and SSIM), Table 2 shows that the degradation mainly resulted from the last small number of convolutional layer(s). For smaller λ values (i.e., λ=∼0.01 to 0.3), the changes in traditional IQ metrics were statistically insignificant.

**Fig. 7:**
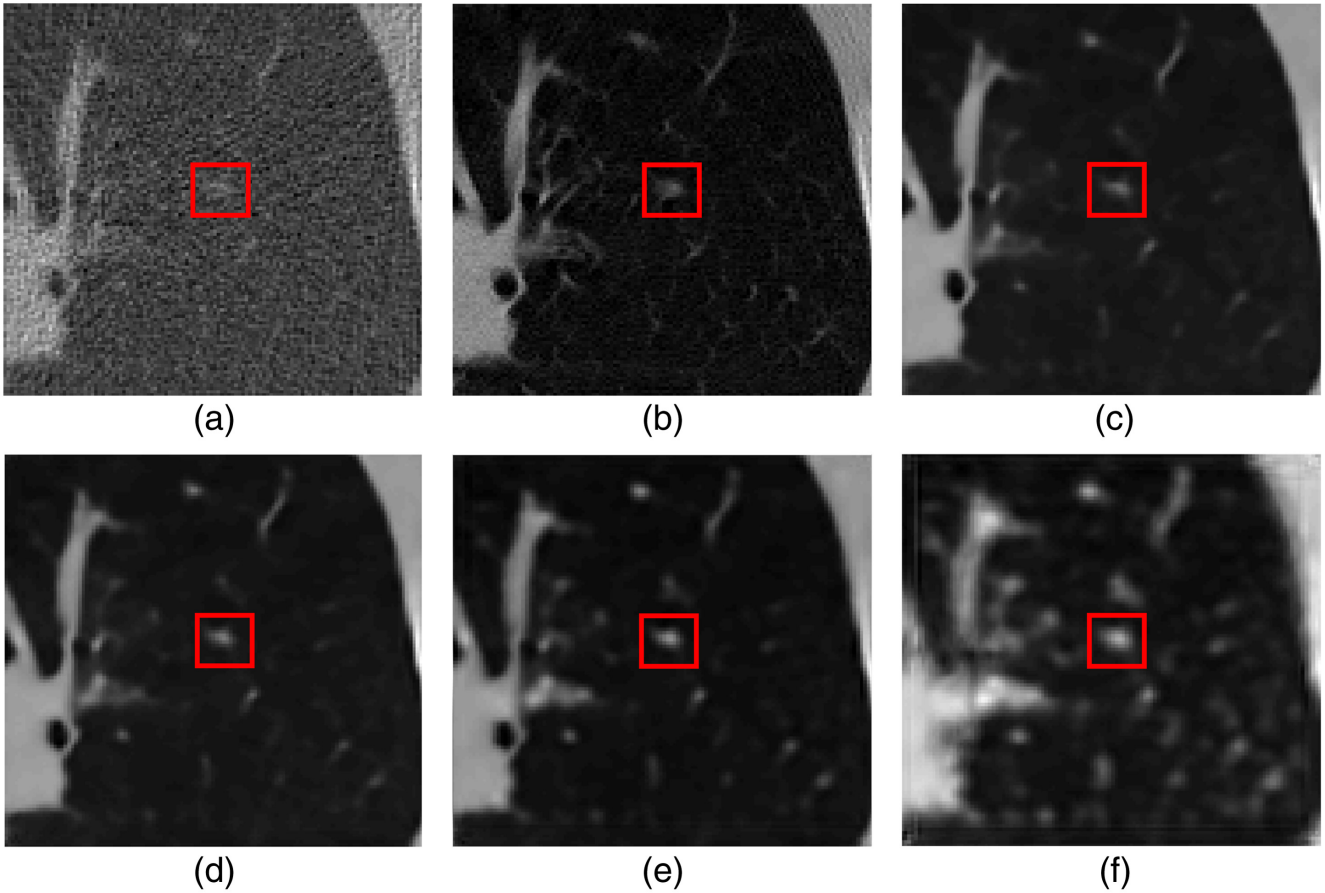
Examples of (a) a low-dose signal-present image, (b) a normal-dose signal-present image, and (c)–(f) images f^ generated by the task-informed denoising network trained using $L_{\text{Hybrid}}$ in which λ=0.01,0.5,0.9,0.99, respectively. The red box indicates the inserted signal. The denoised images estimated by the denoising network that was trained with task-informed loss function $L_{\text{Hybrid}}$ were blurred, and the severity increases along with the increase of λ.

The denoised estimates produced by the task-informed image denoising methods were also subjectively assessed. Figure 7 shows a noisy image and denoised images generated by denoising networks fine-tuned with the hybrid loss for different values of λ in Eq. (8). The denoised estimates were blurred as a result of the task-informed training, and the level of blur increased when λ increased.

#### 5.2.2 Impact of denoising network depth

A study was performed to investigate whether the loss of task-relevant information primarily occurs in the last several layers when the denoising network depths increase. As shown in Table 3, the SLNN-NO performance decreased as a function of denoising network depth, which is consistent with previous findings^9^ that the mantra “deep is better” may not always hold for objective IQ measures. After the task-informed training, the SLNN-NO performance was improved, and the variations of the improved SLNN-NO performance were statistically insignificant when network depth varied. No matter how deep the pretrained denoising network was, the loss of task-relevant information still occurred in the last certain layers (i.e., not related to the depth of denoising networks), at least in the considered cases.

**Table 3:** Relationship between signal detection performance achieved by the SLNN-NO and the depth D of the denoising networks. The SLNN-NO performance on images denoised by the denoising network trained with $L_{\text{Hybrid}}$ and the pretrained non-task-informed denoising networks was quantified. For each of the denoising networks of varying depth, only the last three layers were fine-tuned with $L_{\text{Hybrid}}$. The standard error for AUC values is 0.003. The results indicated that the loss of task-relevant information only occurred in the last (or last several) layer(s), regardless of the depth of the denoising network.

D	9	11	13	15
AUC (Fine-tuned)	0.6984	0.7146	0.7074	0.7130
AUC (Pretrained)	0.5751	0.5714	0.5529	0.5501

### 5.3 Impact of the Task-Shifts for Training and Evaluation

#### 5.3.1 Test cases with different weight parameters λ

**Fig. 8:**
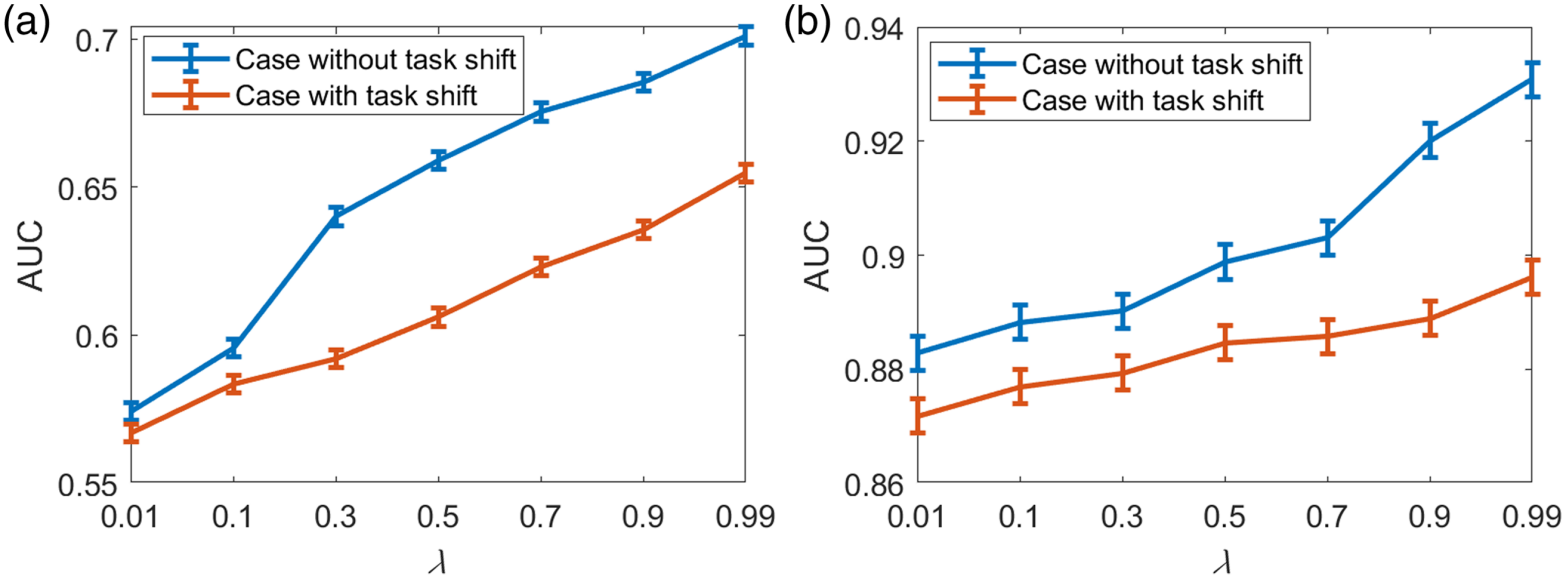
Robustness of the task-informed image denoising method to task-shift assessed for binary signal detection tasks. The SLNN-NO was employed for both model training and objective image quality assessment. The binary signal detection task with random signal locations was considered a complex task, and the binary signal detection task with fixed signal locations was considered a simple task. (a) The red curve represents the task-shift case in which the simple task was used for training and the complex one was used for evaluation. The blue curve represents the reference case without task-shift in which the complex task was used for training and evaluation. (b) The red curve represents the task-shift case in which the complex task was used for training and the simple one was used for evaluation. The blue curve represents the reference case without task-shift in which the simple task was used for training and evaluation. It was observed that using a complex task for denoising model training can mitigate task performance degradation when a task-shift is introduced.

**Fig. 9:**
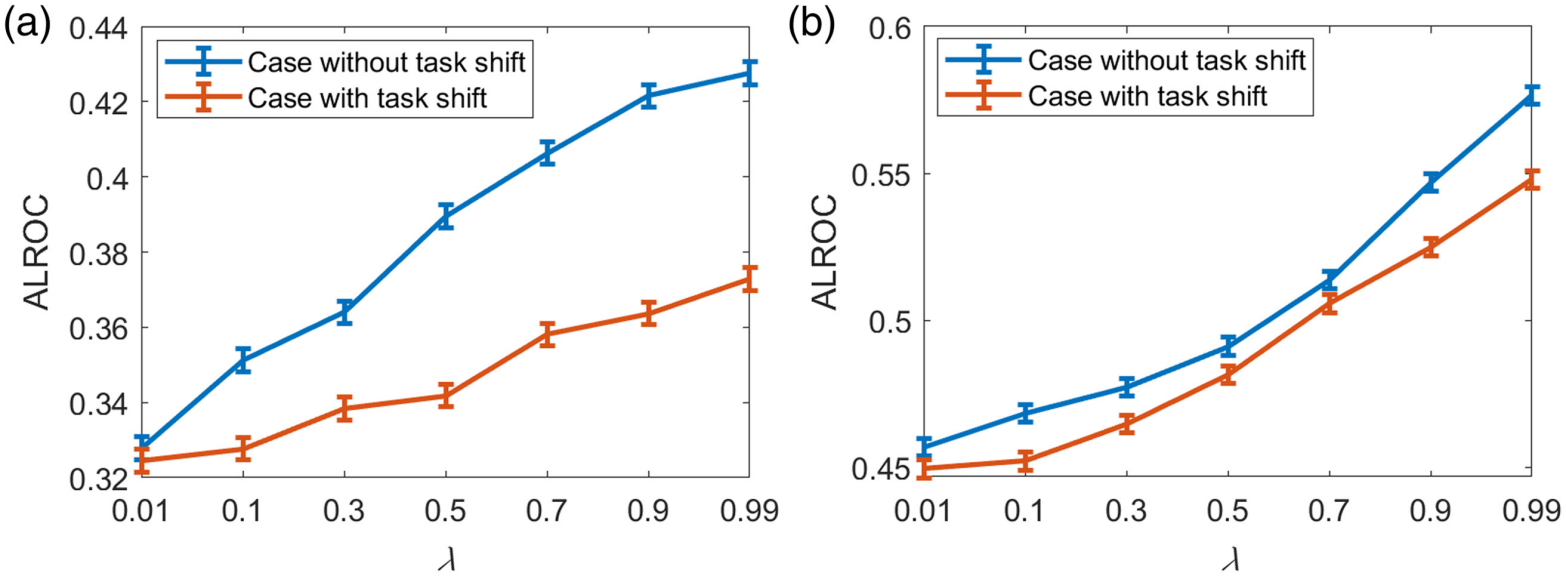
Robustness of the task-informed image denoising method to task-shift assessed for signal detection-localization tasks. The SLNN-NO was employed for both training and objective image quality assessment. The detection-localization task with four possible signal locations was considered a complex task, and the detection-localization task with two possible signal locations was considered a simple task. (a) The red curve represents the task-shift case in which the simple task was used for training and the complex one was used for evaluation. The blue curve represents the reference case without task-shift in which the complex task was used for training and evaluation. (b) The red curve represents the task-shift case in which the complex task was used for training and the simple one was used for evaluation. The blue curve represents the reference case without task-shift in which the simple task was used for training and evaluation. The observations are similar to those in Fig. 8. Using a complex task for denoising model training can mitigate task performance degradation when a task-shift is introduced.

The robustness of the task-informed image denoising method to task-shift was also assessed, and the results are shown in Figs. 8 and 9. The SLNN-NO performance for the case with no task-shift was considered a reference. It was observed that introducing task-shift always degraded the task performance as expected and the degradation resulting from task-shift became insignificant when λ decreased. This is due to the smaller weight for the task-based loss component as λ decreases, which makes the impact of task-shift less significant. For the case in which a relatively simple task was used for training and the complex one was used for evaluation [Figs. 8(a) and 9(a)], the degradation in the task performance was much more significant than in the case in which a complex task was used for training and a simple one was used for evaluation [Figs. 8(b) and 9(b)]. For binary signal detection tasks shown in Fig. 8, this observation is due to the fact that the case with random signal locations can be easily generalized to the case with fixed signal locations but not vice versa.

Similar findings were observed for the detection-localization tasks with both two and four possible signal locations, as shown in Fig. 9. It was found that, when the tasks with four and two possible locations were considered source/target tasks, less significant degradation in the task performance was observed when compared with the case in which the tasks with two and four possible locations were considered source/target tasks. This suggested that employing a relatively complex task for training can better improve the robustness of a task-informed image restoration method to task-shift than employing a simple task.

#### 5.3.2 Test cases with gradually increased mismatches in source/target tasks

**Fig. 10:**
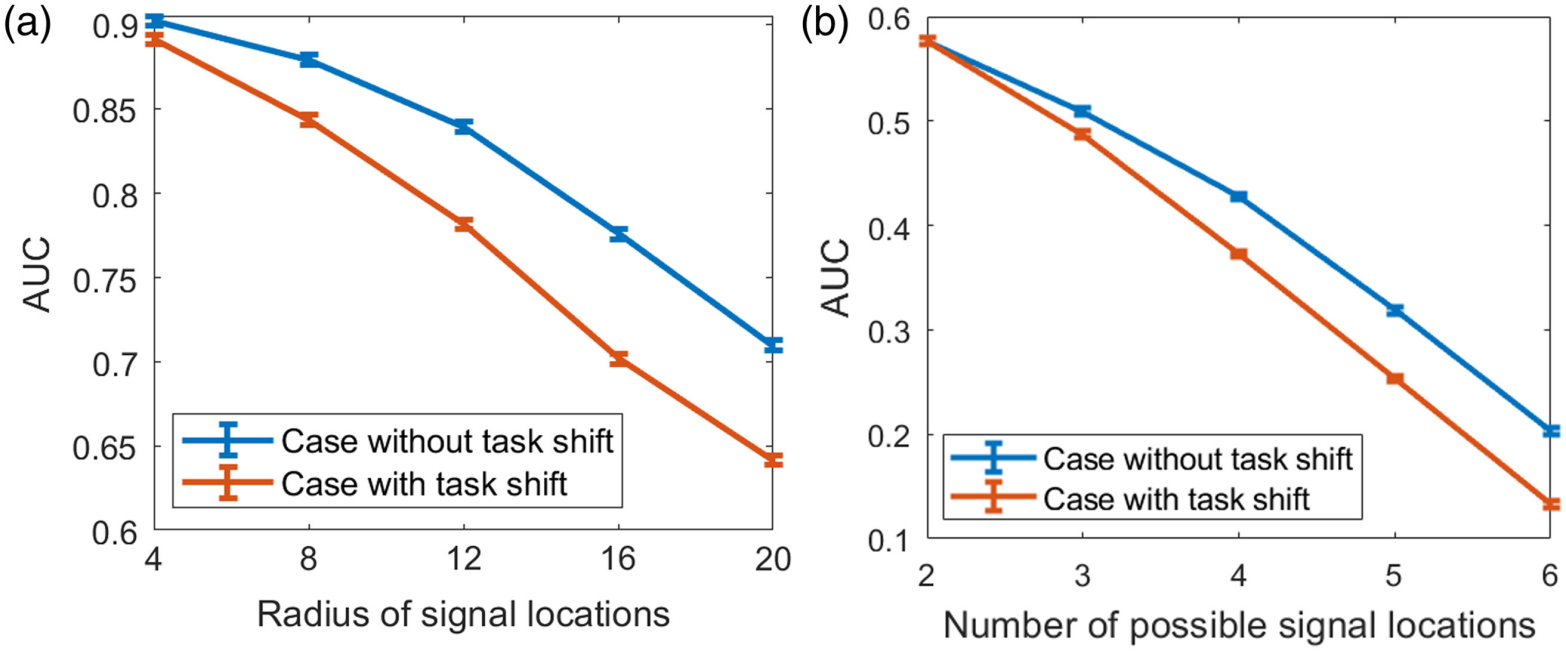
Robustness of the task-informed image denoising method assessed for two cases with gradually increased severities of task-shifts. The SLNN-NO was employed for both denoising network training and objective image quality assessment. (a) In the case of binary signal detection tasks, the source task was a binary signal detection task with a fixed signal location. In the target task, the signal was randomly located within circles with gradually increased radius r={4,8,12,16,20}. (b) In the case of detection-localization tasks, the source task was a task with two possible signal locations, and tasks with {2,3,4,5,6} possible signal locations were considered target tasks. The red curve represents the task-shift case in which the source task was used for training and the target tasks for evaluation. The blue curve represents the reference case in which the target task was considered for denoising network training and performance evaluation. It was observed that, as the task-shift increased, the degradation in task performance became more severe.

Another test case was performed to assess the robustness of the task-informed image denoising method to task-shift, and the results are shown in Fig. 10. The SLNN-NO performance for cases without task-shift was considered a reference. As expected, it was observed that introducing task-shift always degraded the task performance. As shown in Fig. 10(a), for binary signal detection tasks, the SLNN-NO performance decreased as a function of the radius of a circle where the signal was randomly located. In addition, the SLNN-NO performance gap between cases with and without task-shift increased when the radius became larger.

Similar findings were also observed for the detection-localization tasks, as shown in Fig. 10(b). It was observed that the SLNN-NO performance decreased as a function of the number of possible signal locations. In addition, the SLNN-NO performance gap between cases with and without task-shift increased as a function of the number of possible signal locations. This suggests that, when employing the task-informed denoising method, the gap between potential task-shifts between training and evaluation needs to be carefully investigated.

## 6 Discussion and Summary

In this work, a task-informed DNN-based image denoising method that preserves task-specific information was objectively evaluated. This study was motivated by previous works^9^^,^^10^ that indicated that traditional DNN-based denoising methods may not benefit the task performance even though the traditional measures of IQ were improved. The task-informed model training method employed a hybrid loss strategy and only acted on the last several layers of a DNN-based denoising method. To evaluate the method, binary signal detection tasks with fixed and random signal locations under SKS/BKS conditions were considered. The performance of SLNN-NO, SLNN-HO, and common NOs was quantified to assess the impact of task-informed training on task performance preservation.

The numerical results indicated that certain tradeoffs can be achieved such that the resulting AUC value was significantly improved, and the degradation of physical IQ measures was statistically insignificant. The improvement in the signal detection performance of the considered NOs for evaluation can be explained by singular value spectra analysis. It was revealed that the considered task-informed transfer learning approach could mitigate the ill-conditioning of covariance matrices and has the potential to improve the image statistics that are important for signal detection. In addition, it was observed that significant improvements in the task performance were achieved by fine-tuning the last (or last several) convolutional layer(s), whereas the improvement was insignificant when more layers were trainable, which confirmed that the loss of task-relevant information occurred in the last certain layers, at least in the considered cases.

To better understand the potential suitability of a task-informed image restoration method for clinical translation, its robustness to task-shift was also assessed. It was observed that introducing task-shift will degrade the task performance as expected. The degradation was significant when a relatively simple task was considered the source task, whereas a complex one was used as the target task. The degradation can be potentially mitigated by employing the complex task as the source task and the simple one as the target task. This suggests that employing a relatively complex task for training can better improve the robustness of a task-informed image restoration method to task-shift than employing a simple task.

There remain numerous important topics for future investigation. In this work, the SLNN-NO and SLNN-HO were employed to compute the task component $L_{t}$ in Eq. (8). Anthropomorphic numerical observers (ANOs) may instead be employed to predict the human observer performance.^29^^,^^45^^,^^46^ Employing an ANO to compute $L_{t}$ may potentially benefit a task-informed image denoising method if humans are the ultimate readers of the image. The evaluation study in this paper focuses on significant parameters such as the weight parameter λ in Eq. (8) and the number of trainable layers. Other parameters, such as the size of training dataset and the ratio between signal-present and signal-absent images, remain unexplored. The extension of the proposed method for use with more complex tasks such as detection-estimation tasks^32^^,^^47^ is also an important topic. The task-informed image denoising method and the corresponding assessment strategy can also readily be applied to different image restoration and reconstruction methods. Ultimately, it will be critical to conduct human reader studies to assess the benefit of any task-informed method.

## Data Availability

Code and data will be made publicly available upon acceptance of the paper.
